# Health Behaviors and Oral Health Among Centenarians in Nanjing, China: A Cross-Sectional Study

**DOI:** 10.1093/geroni/igaa057.3049

**Published:** 2020-12-16

**Authors:** Xin Xu, Yuan Zhao, Danan Gu, Yaolin Pei, Bei Wu

**Affiliations:** 1 Nanjing Normal University, Nanjing, Jiangsu, China; 2 United Nations, New York, New York, United States; 3 New York University, New York, New York, United States

## Abstract

The aim of this study was to examine the association between health behaviors and oral health among Chinese centenarians. Data from the Nanjing Centenarians Study (NCS) in China was used (N=185, Mean age = 102). Oral health status was measured by self-reported oral health and edentulous status. Results from ordinal regression and logistic regression models indicated that centenarians who were male, smoking, normal weight (18.5-24.9kg/m2), participated in more activities, and brushed teeth more frequently were more likely to report better oral health. Those who ate fruits daily and brushed teeth more frequently were more likely to be dentate. The association between frequent toothbrushing and oral health was stronger for those who had some formal education and were living with family members. Our study demonstrated the significance of health behaviors on oral health in very old age, and the importance of lifestyle on healthy aging.

